# Treatment of uncomplicated UTI in males: a systematic review of the literature

**DOI:** 10.3399/bjgpopen20X101140

**Published:** 2021-02-03

**Authors:** Karen Farrell, Meera Tandan, Virginia Hernandez Santiago, Ildiko Gagyor, Anja Maria Braend, Marius Skow, Ingvild Vik, Filip Jansaaker, Gail Hayward, Akke Vellinga

**Affiliations:** 1 Department of General Practice, HRB Primary Care Clinical Trials Network Ireland, School of Medicine, National University of Ireland, Galway, Ireland; 2 Cecil G Sheps Center for Health Services Research, University of North Carolina at Chapel Hill, Chapel Hill, North Carolina, US; 3 Division of Population and Behavioural Sciences, School of Medicine, University of St Andrews, St Andrews, UK; 4 Department of General Practice, Universitatsklinikum Wurzburg, Wurzburg, Bavaria, Germany; 5 The Antibiotic Centre for Primary Care, Department of General Practice, Institute of Health and Society, University of Oslo, Oslo, Norway; 6 Department of Clinical Microbiology, Rigshospitalet, Kobenhavn, Denmark; 7 Center for Primary Health Care Research, Lund University, Lund, Sweden; 8 Nuffield Department of Primary Care Health Science, University of Oxford, Oxford, UK; 9 School of Medicine, National University of Ireland, Galway, Ireland

**Keywords:** male, urinary tract infections, antibiotic treatment, primary health care, randomised clinical trial, review

## Abstract

**Background:**

Urinary tract infections (UTIs) affect around 20% of the male population in their lifetime. The incidence of UTIs in men in the community is 0.9–2.4 cases per 1000 aged <55 years and 7.7 per 1000 aged ≥85 years.

**Aim:**

To evaluate the outcomes of randomised controlled trials (RCTs) comparing the effectiveness of different antimicrobial treatments and durations for uncomplicated UTIs in adult males in outpatient settings.

**Method:**

A systematic literature review of RCTs of adult male patients with an uncomplicated UTI treated with oral antimicrobials in any outpatient setting. The outcomes were symptom resolution within 2 weeks of starting treatment, duration until symptom resolution, clinical cure, bacteriological cure, and frequency of adverse events.

**Results:**

From the 1052 abstracts screened, three provided sufficient information on outcomes. One study compared trimethoprim-sulfamethoxazole for 14 days (21 males) with 42 days (21 males). Fluoroquinolones were compared in the two other RCTs: lomefloxacin (10 males) with norfloxacin (11 males), and ciprofloxacin for 7 days (19 males) and 14 days (19 males). Combining the results from the three RCTs shows that for 75% males with a UTI (76/101) bacteriological cure was reported at the end of the study. Of the 59 patients receiving a fluoroquinolone, 57 (97%) reported bacteriological and clinical cure within 2 weeks after treatment.

**Conclusion:**

The evidence available is insufficient to make any recommendations in relation to type and duration of antimicrobial treatment for male UTIs. Sufficiently powered RCTs are needed to identify best treatment type and duration for male UTIs in primary care.

## How this fits in

As the prevalence of urinary tract infection is much higher in females, most of the research has focused on this group. Only three RCTs including a total of 101 males compared different antibiotic treatments for UTI. The clear lack of RCTs and evidence of best practice shows the urgent need for sufficiently powered RCTs to identify best treatment and duration for male UTIs.

## Introduction

UTIs affect around 20% of the male population in their lifetime.^1^ Incidence of UTI in the community is 0.9–2.4 cases per 1000 men aged <55 years, and up to 7.7 per 1000 in men aged ≥85 years.^2^ UTIs are a common cause of bacteraemia and recurrent infections in this population.^3,4^ UTIs are the second most common cause for antibiotic use in primary care.^5^

Treatment guidelines for male UTIs vary. In the UK (the National Institute for Health and Care Excellence [NICE]), Ireland (Strategy for the control of Antimicrobial Resistance in Ireland [SARI]), and Scotland (Intercollegiate Guideline Network [SIGN]) the guidelines recommend trimethoprim or nitrofurantoin as a firstline treatment options for 7 days for male UTIs, or pivmecillinam or ciprofloxacin in case of chronic kidney disease (defined by national cut-off points). The use of secondline antibiotics is assessed based on culture results while also considering any alternative diagnosis.^6–8^ This is similar to Denmark, Sweden, Norway, and Germany, where trimethoprim and nitrofurantoin are also the firstline treatment, in addition to pivmecillinam.^9–12^

Diagnosing and treating a male UTI is challenging. This is partly owing to infrequent presentation and limited evidence available for this acute condition,^13^ and GPs often treat these as complicated UTIs ‘to be sure’,^14^ which usually involves the use of secondline antimicrobial agents, or longer courses. Up to now, most literature has been focused on research and optimal prescribing for UTIs in females, where treatment guidelines and duration are more clear cut.^15–18^ Even though there is a low incidence of UTIs in men aged <55 years, incidence in older men is similar to female UTIs, particularly in patients with prostate problems, those with indwelling catheters, and those who are hospitalised or in long-term care facilities.^19,20^

Prescribing guidelines have inconsistent advice on type and duration of antibiotic courses for male UTIs.^21^ Unlike randomised controlled trials (RCTs) on female UTIs,^22^ few RCTs include men with community-acquired UTIs who are treated in primary care.^23^ Symptoms of a typical male UTI include: lower urinary tract irritative symptoms such as urgency, frequency, dysuria, and nocturia.^24^ If not treated timely and appropriately, these symptoms may lead to pyelonephritis (kidney infection), which is characterised by fever, and costovertebral angle tenderness.^25^

This review aims to identify RCTs evaluating the effectiveness and duration of different antimicrobial regimens for uncomplicated UTIs in adult males in an outpatient setting.

## Method

### Data source and search strategy

The Cochrane methodology was adopted to perform a systematic search of the literature.^26^ The search was conducted in MEDLINE, Embase, PubMed and the Cochrane Register of Controlled Trials (CENTRAL) (Cochrane Library and Wiley), and CINAHL (EBSCOhost) from 13 March to 10 April 2019 to identify potentially relevant randomised trials focusing on male UTIs. The search terms included were 'urinary tract infections', 'men', 'male', 'treatment', 'treatment dose', 'duration', 'regimens and therapies', 'antimicrobials', 'antibiotics', 'randomised controlled trial*', 'placebo trials', 'pragmatic trials', and 'RCT'. Further, to simplify the search terms, the following were also searched for: 'urinary tract', 'recurrence', 'uncomplicated', 'acute cystitis for urinary tract infections'; and a specific list of antimicrobials, including 'ciprofloxacin', 'norfloxacin', 'fluoroquinolones', 'nalidixic acid', 'ofloxacin', 'moxifloxacin', 'amoxicillin', 'amoxiclav', 'cephalexin', 'nitrofurantoin', 'fosfomycin', 'trimethoprim', 'trimethoprim/sulfamethoxazole', 'beta-lactam', 'pivampicillin', 'pivmecillinam'. The reference lists of articles identified during the screening process were searched to identify any relevant papers for inclusion.

### Screening and eligibility

All RCTs identified were uploaded to the bibliographic management software (EndNote X9 for Windows). Duplicate studies were removed. All potentially relevant papers identified during the screening process were uploaded to Covidence review software management.^27^ Title, abstract, and full-text screening were completed by two researchers independently and articles that remained unclear were discussed collectively until consensus was reached.

### Inclusion and exclusion criteria

Studies included were trials in adult male patients treated with antimicrobials for UTI that reported outcome data on orally administrated antimicrobials comparing different treatments (antimicrobial with antimicrobial or placebo or no treatment or symptomatic) in outpatient settings.

The exclusion criteria were as follows: conditions not consistent with uncomplicated UTI; setting other than primary care; and prophylactic or pharmacokinetic studies.

### Data extraction

Two reviewers independently extracted data from selected full-text articles for inclusion. A standardised form was developed in Covidence. This included the following: year of publication; study population; study period; study site (country); demographic characteristics and number of participants; type, dose and duration of antimicrobial treatments being compared; number of days until resolution of symptoms; recurrence of symptoms; emergence of resistance; and name and types of adverse events that occurred.

### Study outcomes

Outcome data extracted from the eligible RCTs included:

- Relief of symptoms within 2 weeks of starting treatment (defined as ‘cure’)
- Duration until relief of symptoms
- Relief of symptoms at end of the study (according to study duration time periods: 2 weeks, 30 days, 6 weeks)
- Bacteriological cure
- Recurrence of symptoms (according to study duration time periods: 6 weeks, 5–9 days and 30  days after end of treatment)
- Frequency and type of adverse events
- Antimicrobial resistance (but this was never reported).

### Risk of bias assessment

Quality was assessed by two reviewers independently for each paper using the Cochrane Risk of Bias Tool for Randomised controlled Trials proposed by Higgins *et al*.^28^ Disagreement about particular studies were resolved by discussion to develop consensus; a third reviewer was available when necessary.

## Results

**Figure 1.**
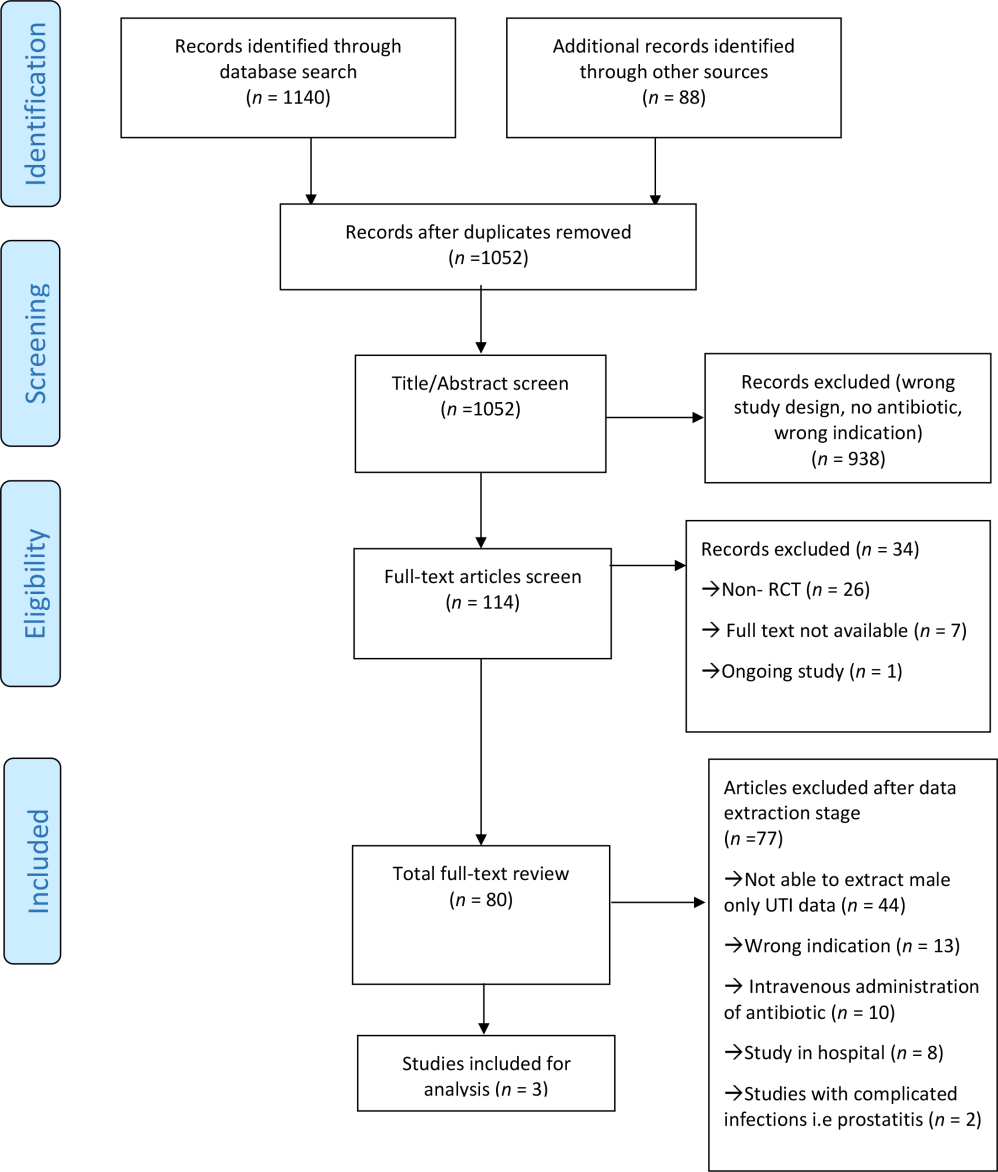
PRISMA flow diagram. RCT = randomised controlled trial; UTI = urinary tract infection.

A total of 1052 titles and abstracts, and 80 full-text papers were reviewed, and three RCTs met the inclusion criteria. Figure 1 represents the PRISMA flow diagram for study inclusion, with full characteristics of included RCTs in Table 1.

**Table 1.** Descriptive characteristics of studies included

**Characteristics/studies**	Gleckman *et al* ^30^	Iravani^31^	van Nieuwkoop *et al* ^29^
**Total study duration (months**)	3	Not available	3
**Total sample**	42	727	200
**Male participants**	42	38 (outcome available for 21)	38
**Median patient age, years**	63	53 and 45	64
**Indication**	Recurrent UTI	Uncomplicated UTI	Febrile UTI
**Antimicrobial used**	TMP-SMX (160/800 mg, twice daily)	Lomefloxacin (400 mg, once daily), norfloxacin (400 mg, twice daily)	Ciprofloxacin (500 mg, twice daily)
**Comorbidities reported**	Yes: diabetes (10)	No	Yes: diabetes (9) urological and heart conditions
**Study setting and country**	Urology outpatient clinic, US	Outpatients in medical centres, US	Primary care centres, the Netherlands

TMP-SMX = trimethoprim-sulfamethoxazole.

An RCT by van Nieuwkoop *et al* compared 7 days with 14 days of oral ciprofloxacin (500 mg, twice daily) in 357 women and men aged ≥18 years with a diagnosis of febrile UTI in 35 primary care centres and seven emergency departments.^29^ Outcomes included clinical and bacteriological cure and recurrence on day 30 (1–2 weeks after the end of therapy). For this review the authors extracted and shared data from 38 men who were treated in primary care.

Gleckman *et al* ^30^ conducted an RCT in 42 men with recurrent UTI presenting at the outpatient clinic of the Boston Veterans Administration Centre. Patients were randomised to 2 weeks or 6 weeks of trimethoprim-sulfamethoxazole (160/800 mg, twice daily).^30^ Outcomes reported were bacteriological cure, relapse (therapeutic failure) and recurrence (new infection) up at any time during the follow-up. Follow-up was 6 weeks, with two weekly cultures after the end of treatment.

The third paper was by Iravani who enrolled 727 adults with uncomplicated UTI into 7–10 days lomefloxacin (once daily) or norfloxacin (twice daily) in 27 centres throughout the US.^31^ Outcomes were clinical and bacteriological cure reported 5–9 days after the end of therapy. Results were reported separately for 38 men enrolled in the study for whom outcome data 6 weeks after the end of treatment was available for 21 participants.

The age of participants was not reported consistently across RCTs. Gleckman *et al* ^30^ reported an overall median age of 60 years. Males in the van Nieuwkoop *et al* ^29^ study had a median age of 71 years and 60 years (overall median 64 years) in each group (7 versus 14 days) and Iravani^31^ reported a median age of 53 years and 45 years in each arm of the RCT.

Comorbidities reported were mainly diabetes, which was reported present in 10 patients by Gleckman *et al* ^30^ and nine with diabetes in the van Nieuwkoop *et al* study^29^ while Iravani^31^ did not report on any comorbidities present.

All outcome data are presented in Table 1; however, the only outcome with sufficient data to allow comparison between RCTs was bacterial cure at the end of therapy.

Table 2 shows an overview of the outcomes of the RCTs. The van Nieuwkoop *et al^29^* study showed 100% bacteriological cure for both durations of ciprofloxacin (7 days versus 14 days) and 90% (17 out of 19) and 100% (19 out of 19) clinical cure for each respectively. Iravani^31^ compared lomefloxacin and norfloxacin and reported 100% (10 out of 10) and 91% (10 out of 11) bacteriological cure and 100% clinical cure for both groups 1 week post-therapy.

**Table 2.** Comparison of UTI clinical and bacteriological cure at the end of treatment, and recurrence

**Studies**	**Antimicrobial**	**Dose^a^**	**Duration**	***n***	**Clinical cure**	**Recurrence**	**Bacteriological cure**
					***n***	**%**	***n***	***%***	***n***	**%**
^30^Gleckman *et al* 1979	TMP-SMX+ Placebo	160/800 mg BD	14 days	21			13	62	6	29
	TMP-SMX	160/800 mg BD	42 days	21			6	29	13	62
^31^Iravani 1992	Lomefloxacin	400 mg QD	7–10 days	10	10	100			10	100
	Norfloxacin	400 mg BD	7–10 days	11	11	100			10	91
^29^van Nieuwkoop *et al* 2017	Ciprofloxacin	500 mg BD	7 days	19	17	90	2	11	19	100
	Ciprofloxacin	500 mg BD	14 days	19	19	100	2	11	18^a^	100

BD = twice a day. TMP-SMX = trimethoprim-sulfamethoxazole. QD = once a day. ^a^One missing urine sample.

Gleckman *et al* ^30^ compared trimethoprim-sulfamethoxazole for 14 days with 42 days with 21 patients in each group, and found all patients had an immediate bacteriological response. In the 14 days group, six (29%) reached bacteriological cure, 13 (62%) had recurrence, while in the six-week group, 13 (62%) and six (29%) were reported for each outcome.

Combining the results from the three RCTs shows that 76 male patients with UTI (out of 101 patients, 75%) reported bacteriological cure at the end of the study. Of the 59 patients receiving a fluoroquinolone, 57 (97%) reported bacteriological and clinical cure within 2 weeks after the end of treatment.

### Adverse events

All three RCTs reported adverse events from antimicrobial treatment, with both Gleckman *et al* ^30^ and van Nieuwkoop *et al* ^29^ reporting separately for male participants. Adverse events were not reported separately for males and females in the Iravani^31^ study. In Gleckman *et al*, two male patients in the 14-day course of trimethoprim group reported five adverse events (chills, sweats and flushing,^1^ transient rash and pruritus^1^), while four adverse events (diffuse urticarial,^1^ nausea and vomiting,^1^ elevated serums creatinine^2^) were reported for four patients in the group receiving 42 days trimethoprim group. In the van Nieuwkoop *et al* ^29^ study, two patients who were treated with ciprofloxacin for 7 days reported to have developed pyelonephritis, and no adverse events were reported in the 14-day ciprofloxacin group.

### Risk of bias assessment

Risk of bias was low overall for the van Nieuwkoop *et al* ^29^ study, while a high risk of bias was determined for the Iravani^31^ study in three domains: blinding of participants, incomplete outcome data, and other sources of bias (unclear timeframe, allocation, outcome assessment, only 5% male). In Gleckman *et al* ^30^ risk of bias was unclear for blinding and allocation concealment, while high risk of bias was documented for incomplete outcome data. Table 3 provides an overview of the risk of bias assessment of the included RCTs.

**Table 3.** Risk of bias assessment

	Gleckman *et al* ^30^	Iravani^31^	van Nieuwkoop *et al* ^29^
**Sequence** **g** **eneration**	Low: table of random digits	Low	Low: randomised stratified per centre and sex. Computer-generated randomisation list
**Allocation** **c** **oncealment**	Unclear: no information provided	No comment	Low: treatment allocation completed after urine culture results. Restricted access to independent pharmacy
**Blinding of participants and personnel for all outcomes**	Unclear: no description of the blinding provided	High: no blinding of the participants or personnel only investigators	Low: double blinding
**Blinding of outcome assessors for all outcomes**	Unclear: no description of the blinding provided	Low: investigators were blinded through third party. The drugs were dispensed by an independent third party to ensure investigator blinding	Low: analysis based on intention to treat population
**Incomplete outcome data for all outcomes**	High: data not reported for two patients in each group suffering adverse events	High: the main outcome (clinical recovery) is reported for 436/727 patients only	Low
**Selective outcome reporting**	Low	Unclear: both outcomes assessed were reported, but no pre-published protocol is available to control this with the initial design	Unclear: all outcomes described in methods chapter are reported
**Other sources of bias**	Low	High: men are just 5% of population and a subgroup of the study. Dropout is about 50% for bacteriological cure and unclear for clinical cure.	None identified

## Discussion

### Summary

This review identified three RCTs evaluating the effectiveness and duration of different antimicrobial regimens for uncomplicated UTIs in adult males. Only three papers met the eligibility criteria after full-text screening and were included in the review, two of which are over 20 years old (Gleckman *et al* from 1979^30^ and Iravani from 1992^31^).

Iravani^31^ and van Nieuwkoop *et al* ^29^ included both male and female patients, but male-only data was obtained from van Nieuwkoop *et al* and could be extracted from Iravani’s paper. Both RCTs compared a fluoroquinolone (ciprofloxacin or lomefloxacin and norfloxacin) for a course of 7–14 days and observed at least 97% clinical and bacteriological cure within 2 weeks. However, the total samples size comparing fluoroquinolones is only 59 and is not sufficient to draw any conclusions. The Gleckman *et al* article focused solely on male participants, and was the only study to use trimethoprim to treat male UTIs. Bacteriological cure was reached in 29% of the 2-week treatment group, while the 6-week group reported 62% at the end of the study. One of the differences with the other RCTs was that Gleckman *et al* included male patients with a recurrent UTI, but it is difficult to ascertain how this may affect the outcomes or the reliability of the findings because of the small sample size. There is clearly a lack of RCTs to allow comparison of findings, especially in relation to trimethoprim. This is particularly concerning as trimethoprim is the firstline recommendation according to the NICE,^6^ SIGN,^7^ and Danish guidelines.^10^ Considering the age of the paper and its limited sample size, it emphasises the need for more up-to-date and verifiable evidence. Furthermore, no RCTs are available comparing any of the other types and durations of firstline treatments, such as nitrofurantoin or pivmecillinam. The use of fluoroquinolones as done in the other two RCTs, which is only recommended as secondline or for complicated infections, suggests that many GPs consider male UTIs complicated, which may result in suboptimal antibiotic choices or longer courses.

### Strengths and limitations

There is a lack of RCTs comparing antimicrobial treatment options and duration for male UTIs, and most RCTs in this population cover complicated UTIs,^32^ asymptomatic bacteriuria, mixed and recurrent infections,^13,33^ and infections in patients with spinal cord injury,^34^ generally in the hospital setting.^19^

Apart from the lack of evidence to identify the best treatment for male patients presenting with UTI to primary care, the present review also shows the absence of relevant patient outcomes. Duration of symptoms, a relevant outcome for patients, is not reported across groups and RCTs, and no inference about duration until clinical or bacteriological cure could be made.

Owing to low sample size, information on adverse events, even though reported, is not sufficient to make conclusions in relation to type or duration of treatment. However, the variability and extent of adverse events reported in patients treated with trimethoprim in Gleckman *et al* ^30^ is noteworthy, but should not be overstated as it may just reflect good reporting rather than higher risk of adverse events. Pyelonephritis was a serious adverse event reported by two out of 19 patients treated with 7-day ciprofloxacin^29^ and would reflect good reporting as no conclusion can be based on this owing to the low numbers.

### Comparison with existing literature

In a recent systematic review and meta-analysis of RCTs comparing long-term antibiotics for prevention of recurrent UTI in older adults, no RCTs could be identified that compared treatments in the male population.^35^ An observational study by Montelin *et al* ^36^ assessed nitrofurantoin and pivmecillinam for lower UTIs in men, and included patients treated with trimethoprim for comparison. No difference in any clinical outcome was observed between the three antibiotics prescribed.^36^ Similarly, a recent Danish study comparing treatment durations of pivmecillinam in men, suggested that 5 days with pivmecillinam (400 mg, three times a day) is sufficient in male UTI.^37^ These studies were retrospective and should ideally be repeated as a prospective RCT. Interestingly, a register study of male prescribing from Norway found that even though fluoroquinolones and cefalexin were associated with lower antibiotic switch rates than the recommended firstline UTI antibiotics (pivmecillinam, nitrofurantoin, and trimethoprim), the occurrence of switching was so low (7%) that the current guidelines were considered to be safe.^38^

### Implications for research and practice

From this review, it is clear there is a need for larger and more comprehensive RCTs that include improved diagnosis of male UTI, comparison of different types of treatment, as well as their duration and detail of the antimicrobial resistance of the isolated uropathogens. Improved outcome measures, including patient relevant outcomes such as duration of symptoms, should include the recording of symptom scores, which would also improve the understanding of treatment and diagnosis of male UTI. As male UTIs are less frequent, to be able to do such a trial, multiple countries and settings could be included to provide a sufficient sample size and improve the treatment of male UTI.

In conclusion, the evidence available is insufficient to make any recommendations in relation to type and duration of antimicrobial treatment for male UTI. Sufficiently powered RCTs are needed to improve knowledge of male UTI and to identify the best treatment regimen for this population in primary care.
